# (3*E*,5*E*)-1-Allyl-3,5-bis(4-meth­oxy­benzyl­idene)­piperidin-4-one

**DOI:** 10.1107/S1600536813015195

**Published:** 2013-06-12

**Authors:** Abdulrahman I. Almansour, Raju Suresh Kumar, Natarajan Arumugam, R. Vishnupriya, J. Suresh

**Affiliations:** a Department of Chemistry, College of Sciences, King Saud University, PO Box 2455, Riyadh 11451, Saudi Arabia; b Department of Physics, The Madura College, Madurai 625 011, India

## Abstract

The piperidine ring in the title compound, C_24_H_25_NO_3_, adopts an envelope conformation with the N atom being the flap atom, and each C=C double bond exhibits an *E* conformation. In the crystal, C—H⋯O hydrogen bonds link the mol­ecules, forming supramolecular layers that stack along the *a* axis.

## Related literature

For background to piperidine ring systems, see: Guengerich *et al.* (1973 ▶); Puder *et al.* (2000 ▶). For the biological importance of the title compound, see: Dimmock, Elias *et al.* (1999 ▶); Dimmock, Kandepu *et al.* (1999 ▶). For a similar structure, see: Suresh *et al.* (2007 ▶). For ring conformation analysis, see: Cremer & Pople (1975 ▶).
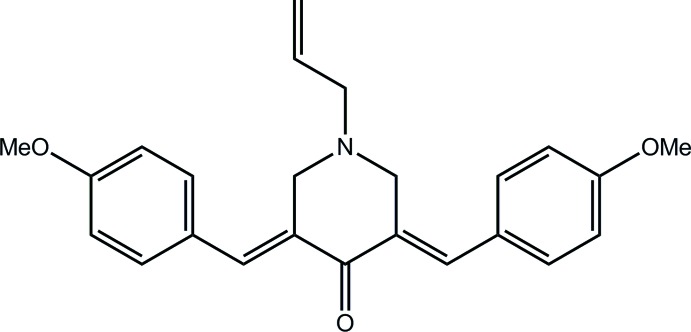


## Experimental

### 

#### Crystal data

C_24_H_25_NO_3_
*M* *_r_* = 375.45
Monoclinic, 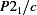

*a* = 19.2409 (15) Å
*b* = 6.8457 (6) Å
*c* = 15.6393 (13) Å
β = 98.255 (2)°
*V* = 2038.6 (3) Å^3^
*Z* = 4
Mo *K*α radiation
μ = 0.08 mm^−1^
*T* = 293 K
0.34 × 0.33 × 0.21 mm

#### Data collection

Bruker Kappa APEXII diffractometer
Absorption correction: multi-scan (*SADABS*; Sheldrick, 1996 ▶) *T*_min_ = 0.973, *T*_max_ = 0.984
22074 measured reflections
5935 independent reflections
3680 reflections with *I* > 2σ(*I*)
*R*_int_ = 0.042

#### Refinement

*R*[*F* ^2^ > 2σ(*F* ^2^)] = 0.050
*wR*(*F* ^2^) = 0.169
*S* = 1.03
5935 reflections
254 parameters
H-atom parameters constrained
Δρ_max_ = 0.19 e Å^−3^
Δρ_min_ = −0.21 e Å^−3^

### 

Data collection: *APEX2* (Bruker, 2004 ▶); cell refinement: *SAINT* (Bruker, 2004 ▶); data reduction: *SAINT*; program(s) used to solve structure: *SHELXS97* (Sheldrick, 2008 ▶); program(s) used to refine structure: *SHELXL97* (Sheldrick, 2008 ▶); molecular graphics: *PLATON* (Spek, 2009 ▶); software used to prepare material for publication: *SHELXL97*.

## Supplementary Material

Crystal structure: contains datablock(s) global, I. DOI: 10.1107/S1600536813015195/tk5227sup1.cif

Structure factors: contains datablock(s) I. DOI: 10.1107/S1600536813015195/tk5227Isup2.hkl

Supplementary material file. DOI: 10.1107/S1600536813015195/tk5227Isup3.cml

Additional supplementary materials:  crystallographic information; 3D view; checkCIF report

## Figures and Tables

**Table 1:** Hydrogen-bond geometry (Å, °)

*D*—H⋯*A*	*D*—H	H⋯*A*	*D*⋯*A*	*D*—H⋯*A*
C10—H10*A*⋯O3^i^	0.97	2.59	3.3710 (18)	138
C21—H21*C*⋯O2^ii^	0.96	2.54	3.466 (2)	163

Symmetry codes: (i) 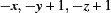; (ii) 
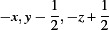
.

**Fig. 1.**
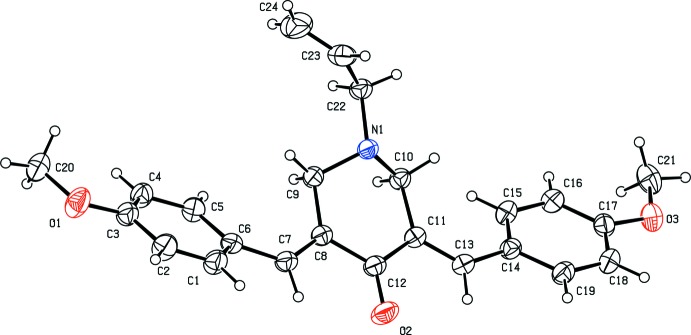
The molecular structure of (I), showing 30% probability displacement ellipsoids and the atom-numbering scheme.

## supplementary crystallographic information

### Comment

Piperidine ring systems are of immense interest in the pharmaceutical industry as they exhibit a wide range of biological activities (Guengerich *et al.*, 1973; Puder *et al.*, 2000). A number of alpha,beta-unsaturated ketones display cytotoxic and anti-cancer properties (Dimmock, Elias *et al.*, 1999; Dimmock, Kandepu *et al.*, 1999) besides being useful synthons for the construction of diverse structurally complex heterocycles. The biological importance of these heterocycles in conjunction with our research interests (Suresh *et al.*, 2007), prompted us to synthesize and report the X-ray studies of the title compound.

In the title compound (Fig 1), the six-membered piperidone ring adopts a sofa conformation which is evidenced by the puckering parameters: q_2_ = 0.5517 (16) Å, θ = 123.96 (5)°, φ = 178 (6)° (Cremer & Pople, 1975). Both olefinic double bonds have an *E* configuration, and the aryl rings are not coplanar with either the adjacent olefinic double bonds or the planar portion of the piperidone ring. The aryl rings are rotated to move atoms C5 and C15 from the plane of the other five atoms of the piperidone ring in the opposite direction of the displacement of atom N1. As the result the torsion angles C5—C6—C7—C8 and C10—C11—C13—C14 have values 31.9 (2) and -4.2 (2)° respectively. This lack of co-planarity is caused by non-bonded interactions between one of the *ortho*-H atoms in the aryl ring and the equatorial H atoms at the 2- and 6- positions of the piperidone ring (H5A/H9A or H9B and H15A/H10A or H10B). These steric repulsions are reduced by the expansion of the bond angle C6—C7—C8 and C11—C13—C14 which are 130.23 (19) and 130.67 (2)° respectively (otherwise 120°).

The C10—H10A···O3 hydrogen bond connect two molecules forming an inverse related dimers which are interlinked by C21—H21C···O2 intermolecular hydrogen bonds to form a supramolecular layer in the *bc* plane.

### Experimental

A equimolar mixture of (3*E*,*5E*)-3,5-bis(4-methoxybenzylidene)piperidin-4-one (0.023 g), allyl chloride (0.100 g) and K_2_CO_3_ (0.041 g) in acetone (30 ml) was stirred at room temperature for 30 minutes. After completion of the reaction as evident from TLC, the excess solvent was removed under vacuum and the crude product was extracted with ethyl acetate and recrystallized from the same to afford the title compound. *M*. pt: 270–272 K. Yield: 92%.

### Refinement

H atoms were placed at calculated positions and allowed to ride on their carrier atoms with C—H = 0.93–0.97 Å, and with *U*_iso_ = 1.2–1.5*U*_eq_(C).

### Crystal data

**Table:**

C_24_H_25_NO_3_	*F*(000) = 800
*M**_r_* = 375.45	*D*_x_ = 1.223 Mg m^−^^3^
Monoclinic, *P*2_1_/*c*	Mo *K*α radiation, λ = 0.71073 Å
Hall symbol: -P 2ybc	Cell parameters from 2000 reflections
*a* = 19.2409 (15) Å	θ = 2–30°
*b* = 6.8457 (6) Å	µ = 0.08 mm^−^^1^
*c* = 15.6393 (13) Å	*T* = 293 K
β = 98.255 (2)°	Block, colourless
*V* = 2038.6 (3) Å^3^	0.34 × 0.33 × 0.21 mm
*Z* = 4	

### Data collection

**Table:**

Bruker Kappa APEXII diffractometer	5935 independent reflections
Radiation source: fine-focus sealed tube	3680 reflections with *I* > 2σ(*I*)
Graphite monochromator	*R*_int_ = 0.042
Detector resolution: 0 pixels mm^-1^	θ_max_ = 30.0°, θ_min_ = 2.1°
ω and φ scans	*h* = −27→27
Absorption correction: multi-scan (*SADABS*; Sheldrick, 1996)	*k* = −9→9
*T*_min_ = 0.973, *T*_max_ = 0.984	*l* = −22→22
22074 measured reflections	

### Refinement

**Table:**

Refinement on *F*^2^	Primary atom site location: structure-invariant direct methods
Least-squares matrix: full	Secondary atom site location: difference Fourier map
*R*[*F*^2^ > 2σ(*F*^2^)] = 0.050	Hydrogen site location: inferred from neighbouring sites
*wR*(*F*^2^) = 0.169	H-atom parameters constrained
*S* = 1.03	*w* = 1/[σ^2^(*F*_o_^2^) + (0.0905*P*)^2^ + 0.0856*P*] where *P* = (*F*_o_^2^ + 2*F*_c_^2^)/3
5935 reflections	(Δ/σ)_max_ < 0.001
254 parameters	Δρ_max_ = 0.19 e Å^−^^3^
0 restraints	Δρ_min_ = −0.21 e Å^−^^3^

### Special details

**Table:**

Geometry. All e.s.d.'s (except the e.s.d. in the dihedral angle between two l.s. planes) are estimated using the full covariance matrix. The cell e.s.d.'s are taken into account individually in the estimation of e.s.d.'s in distances, angles and torsion angles; correlations between e.s.d.'s in cell parameters are only used when they are defined by crystal symmetry. An approximate (isotropic) treatment of cell e.s.d.'s is used for estimating e.s.d.'s involving l.s. planes.
Refinement. Refinement of *F*^2^ against ALL reflections. The weighted *R*-factor *wR* and goodness of fit *S* are based on *F*^2^, conventional *R*-factors *R* are based on *F*, with *F* set to zero for negative *F*^2^. The threshold expression of *F*^2^ > σ(*F*^2^) is used only for calculating *R*-factors(gt) *etc*. and is not relevant to the choice of reflections for refinement. *R*-factors based on *F*^2^ are statistically about twice as large as those based on *F*, and *R*- factors based on ALL data will be even larger.

### Fractional atomic coordinates and isotropic or equivalent isotropic displacement parameters (Å^2^)

**Table:**

	*x*	*y*	*z*	*U*_iso_*/*U*_eq_	
O1	0.54889 (6)	0.02740 (19)	0.11269 (10)	0.0792 (4)	
O2	0.20688 (7)	0.68527 (18)	0.21213 (10)	0.0775 (4)	
O3	−0.14813 (6)	0.6458 (2)	0.45644 (8)	0.0682 (3)	
N1	0.21732 (5)	0.20325 (16)	0.35611 (7)	0.0421 (3)	
C1	0.43254 (8)	0.4039 (2)	0.16588 (11)	0.0598 (4)	
H1A	0.4346	0.5385	0.1740	0.072*	
C2	0.49119 (8)	0.3075 (3)	0.14716 (13)	0.0651 (5)	
H2A	0.5325	0.3761	0.1444	0.078*	
C3	0.48852 (8)	0.1090 (2)	0.13251 (10)	0.0550 (4)	
C4	0.42736 (8)	0.0081 (2)	0.13796 (10)	0.0563 (4)	
H4A	0.4252	−0.1257	0.1277	0.068*	
C5	0.36911 (7)	0.1063 (2)	0.15869 (10)	0.0526 (4)	
H5A	0.3283	0.0364	0.1631	0.063*	
C6	0.37000 (7)	0.3065 (2)	0.17309 (9)	0.0473 (3)	
C7	0.31027 (7)	0.4208 (2)	0.19275 (9)	0.0501 (3)	
H7A	0.3086	0.5484	0.1722	0.060*	
C8	0.25757 (7)	0.3710 (2)	0.23565 (9)	0.0445 (3)	
C9	0.25089 (7)	0.1769 (2)	0.27873 (9)	0.0449 (3)	
H9A	0.2230	0.0889	0.2390	0.054*	
H9B	0.2970	0.1196	0.2945	0.054*	
C10	0.14497 (7)	0.2688 (2)	0.33075 (9)	0.0438 (3)	
H10A	0.1205	0.2705	0.3809	0.053*	
H10B	0.1206	0.1791	0.2887	0.053*	
C11	0.14472 (6)	0.4704 (2)	0.29240 (9)	0.0430 (3)	
C12	0.20337 (7)	0.5227 (2)	0.24397 (10)	0.0493 (3)	
C13	0.09767 (7)	0.6104 (2)	0.30150 (9)	0.0456 (3)	
H13A	0.1067	0.7300	0.2770	0.055*	
C14	0.03457 (7)	0.6064 (2)	0.34352 (8)	0.0448 (3)	
C15	−0.00436 (7)	0.4391 (2)	0.35423 (9)	0.0485 (3)	
H15A	0.0109	0.3196	0.3356	0.058*	
C16	−0.06544 (7)	0.4458 (2)	0.39197 (9)	0.0510 (3)	
H16A	−0.0906	0.3320	0.3983	0.061*	
C17	−0.08852 (7)	0.6221 (2)	0.42007 (9)	0.0511 (4)	
C18	−0.05055 (9)	0.7910 (2)	0.41030 (11)	0.0601 (4)	
H18A	−0.0656	0.9099	0.4297	0.072*	
C19	0.00918 (8)	0.7825 (2)	0.37195 (10)	0.0550 (4)	
H19A	0.0335	0.8973	0.3647	0.066*	
C20	0.55156 (11)	−0.1755 (3)	0.10006 (14)	0.0770 (5)	
H20A	0.5972	−0.2110	0.0871	0.116*	
H20B	0.5165	−0.2125	0.0529	0.116*	
H20C	0.5429	−0.2415	0.1516	0.116*	
C21	−0.18746 (8)	0.4758 (3)	0.47126 (12)	0.0704 (5)	
H21A	−0.2279	0.5130	0.4971	0.106*	
H21B	−0.1586	0.3890	0.5094	0.106*	
H21C	−0.2024	0.4110	0.4173	0.106*	
C22	0.21975 (7)	0.0227 (2)	0.40710 (10)	0.0496 (3)	
H22A	0.2046	−0.0860	0.3692	0.060*	
H22B	0.1875	0.0335	0.4491	0.060*	
C23	0.29171 (9)	−0.0170 (3)	0.45282 (11)	0.0654 (4)	
H23A	0.3125	0.0780	0.4907	0.079*	
C24	0.32751 (11)	−0.1738 (4)	0.44379 (16)	0.0994 (8)	
H24C	0.3085	−0.2720	0.4065	0.119*	
H24A	0.3723	−0.1884	0.4746	0.119*	

### Atomic displacement parameters (Å^2^)

**Table:**

	*U*^11^	*U*^22^	*U*^33^	*U*^12^	*U*^13^	*U*^23^
O1	0.0652 (7)	0.0575 (8)	0.1236 (11)	0.0088 (6)	0.0433 (7)	0.0043 (7)
O2	0.0787 (8)	0.0532 (7)	0.1092 (10)	0.0193 (6)	0.0431 (7)	0.0334 (7)
O3	0.0634 (6)	0.0774 (9)	0.0692 (7)	0.0096 (6)	0.0277 (6)	−0.0027 (6)
N1	0.0428 (5)	0.0382 (6)	0.0462 (6)	0.0033 (5)	0.0093 (4)	0.0052 (5)
C1	0.0638 (9)	0.0407 (9)	0.0803 (11)	−0.0020 (7)	0.0286 (8)	0.0058 (7)
C2	0.0574 (9)	0.0503 (10)	0.0933 (13)	−0.0061 (7)	0.0308 (8)	0.0056 (9)
C3	0.0543 (8)	0.0500 (9)	0.0641 (9)	0.0048 (7)	0.0202 (7)	0.0044 (7)
C4	0.0624 (9)	0.0424 (8)	0.0669 (9)	−0.0012 (7)	0.0186 (7)	−0.0032 (7)
C5	0.0501 (7)	0.0487 (9)	0.0602 (9)	−0.0052 (6)	0.0125 (6)	−0.0022 (7)
C6	0.0506 (7)	0.0458 (8)	0.0473 (7)	0.0027 (6)	0.0132 (6)	0.0075 (6)
C7	0.0537 (7)	0.0438 (8)	0.0548 (8)	0.0043 (6)	0.0151 (6)	0.0097 (6)
C8	0.0468 (7)	0.0404 (8)	0.0469 (7)	0.0023 (6)	0.0085 (5)	0.0032 (6)
C9	0.0460 (6)	0.0404 (7)	0.0498 (7)	0.0026 (6)	0.0123 (5)	0.0021 (6)
C10	0.0398 (6)	0.0419 (7)	0.0503 (7)	0.0016 (5)	0.0080 (5)	0.0021 (6)
C11	0.0417 (6)	0.0404 (7)	0.0461 (7)	0.0020 (5)	0.0035 (5)	0.0012 (6)
C12	0.0511 (7)	0.0435 (8)	0.0547 (8)	0.0061 (6)	0.0122 (6)	0.0088 (7)
C13	0.0456 (6)	0.0413 (7)	0.0491 (7)	0.0031 (6)	0.0040 (5)	0.0037 (6)
C14	0.0443 (6)	0.0434 (8)	0.0454 (7)	0.0058 (6)	0.0014 (5)	0.0006 (6)
C15	0.0427 (6)	0.0442 (8)	0.0576 (8)	0.0043 (6)	0.0034 (6)	−0.0060 (6)
C16	0.0466 (7)	0.0492 (9)	0.0565 (8)	−0.0003 (6)	0.0049 (6)	−0.0011 (7)
C17	0.0488 (7)	0.0611 (10)	0.0437 (7)	0.0097 (7)	0.0078 (6)	−0.0007 (7)
C18	0.0688 (9)	0.0478 (9)	0.0662 (10)	0.0134 (8)	0.0176 (8)	−0.0034 (7)
C19	0.0610 (8)	0.0414 (8)	0.0643 (9)	0.0052 (7)	0.0145 (7)	0.0023 (7)
C20	0.0843 (12)	0.0616 (12)	0.0891 (14)	0.0203 (10)	0.0259 (10)	−0.0022 (10)
C21	0.0566 (9)	0.0971 (15)	0.0604 (9)	−0.0052 (9)	0.0180 (7)	−0.0085 (10)
C22	0.0515 (7)	0.0427 (8)	0.0566 (8)	0.0023 (6)	0.0141 (6)	0.0092 (6)
C23	0.0619 (9)	0.0723 (12)	0.0616 (9)	0.0051 (9)	0.0075 (7)	0.0234 (9)
C24	0.0765 (12)	0.1123 (19)	0.1129 (17)	0.0407 (13)	0.0262 (12)	0.0450 (15)

### Geometric parameters (Å, º)

**Table:**

O1—C3	1.3639 (18)	C11—C13	1.3403 (19)
O1—C20	1.405 (2)	C11—C12	1.4902 (19)
O2—C12	1.2253 (18)	C13—C14	1.4614 (19)
O3—C17	1.3611 (17)	C13—H13A	0.9300
O3—C21	1.425 (2)	C14—C15	1.392 (2)
N1—C10	1.4623 (16)	C14—C19	1.397 (2)
N1—C9	1.4624 (17)	C15—C16	1.3896 (19)
N1—C22	1.4681 (18)	C15—H15A	0.9300
C1—C2	1.375 (2)	C16—C17	1.380 (2)
C1—C6	1.394 (2)	C16—H16A	0.9300
C1—H1A	0.9300	C17—C18	1.388 (2)
C2—C3	1.378 (2)	C18—C19	1.372 (2)
C2—H2A	0.9300	C18—H18A	0.9300
C3—C4	1.378 (2)	C19—H19A	0.9300
C4—C5	1.385 (2)	C20—H20A	0.9600
C4—H4A	0.9300	C20—H20B	0.9600
C5—C6	1.389 (2)	C20—H20C	0.9600
C5—H5A	0.9300	C21—H21A	0.9600
C6—C7	1.4589 (19)	C21—H21B	0.9600
C7—C8	1.3379 (19)	C21—H21C	0.9600
C7—H7A	0.9300	C22—C23	1.489 (2)
C8—C12	1.4906 (19)	C22—H22A	0.9700
C8—C9	1.5033 (19)	C22—H22B	0.9700
C9—H9A	0.9700	C23—C24	1.295 (3)
C9—H9B	0.9700	C23—H23A	0.9300
C10—C11	1.5040 (19)	C24—H24C	0.9300
C10—H10A	0.9700	C24—H24A	0.9300
C10—H10B	0.9700		
			
C3—O1—C20	119.09 (14)	C11—C12—C8	117.87 (12)
C17—O3—C21	118.02 (13)	C11—C13—C14	130.67 (13)
C10—N1—C9	109.25 (11)	C11—C13—H13A	114.7
C10—N1—C22	111.15 (10)	C14—C13—H13A	114.7
C9—N1—C22	111.25 (11)	C15—C14—C19	116.93 (13)
C2—C1—C6	122.13 (15)	C15—C14—C13	124.46 (13)
C2—C1—H1A	118.9	C19—C14—C13	118.56 (13)
C6—C1—H1A	118.9	C16—C15—C14	121.79 (14)
C1—C2—C3	119.80 (14)	C16—C15—H15A	119.1
C1—C2—H2A	120.1	C14—C15—H15A	119.1
C3—C2—H2A	120.1	C17—C16—C15	119.64 (15)
O1—C3—C4	124.88 (15)	C17—C16—H16A	120.2
O1—C3—C2	115.40 (14)	C15—C16—H16A	120.2
C4—C3—C2	119.72 (14)	O3—C17—C16	124.50 (15)
C3—C4—C5	119.90 (15)	O3—C17—C18	115.81 (14)
C3—C4—H4A	120.1	C16—C17—C18	119.68 (13)
C5—C4—H4A	120.1	C19—C18—C17	120.02 (15)
C4—C5—C6	121.71 (13)	C19—C18—H18A	120.0
C4—C5—H5A	119.1	C17—C18—H18A	120.0
C6—C5—H5A	119.1	C18—C19—C14	121.94 (15)
C5—C6—C1	116.72 (13)	C18—C19—H19A	119.0
C5—C6—C7	124.86 (13)	C14—C19—H19A	119.0
C1—C6—C7	118.41 (14)	O1—C20—H20A	109.5
C8—C7—C6	130.23 (14)	O1—C20—H20B	109.5
C8—C7—H7A	114.9	H20A—C20—H20B	109.5
C6—C7—H7A	114.9	O1—C20—H20C	109.5
C7—C8—C12	117.16 (13)	H20A—C20—H20C	109.5
C7—C8—C9	124.73 (12)	H20B—C20—H20C	109.5
C12—C8—C9	118.06 (11)	O3—C21—H21A	109.5
N1—C9—C8	109.79 (11)	O3—C21—H21B	109.5
N1—C9—H9A	109.7	H21A—C21—H21B	109.5
C8—C9—H9A	109.7	O3—C21—H21C	109.5
N1—C9—H9B	109.7	H21A—C21—H21C	109.5
C8—C9—H9B	109.7	H21B—C21—H21C	109.5
H9A—C9—H9B	108.2	N1—C22—C23	111.66 (12)
N1—C10—C11	109.77 (10)	N1—C22—H22A	109.3
N1—C10—H10A	109.7	C23—C22—H22A	109.3
C11—C10—H10A	109.7	N1—C22—H22B	109.3
N1—C10—H10B	109.7	C23—C22—H22B	109.3
C11—C10—H10B	109.7	H22A—C22—H22B	107.9
H10A—C10—H10B	108.2	C24—C23—C22	124.8 (2)
C13—C11—C12	117.03 (13)	C24—C23—H23A	117.6
C13—C11—C10	125.27 (12)	C22—C23—H23A	117.6
C12—C11—C10	117.63 (11)	C23—C24—H24C	120.0
O2—C12—C11	120.98 (13)	C23—C24—H24A	120.0
O2—C12—C8	121.15 (13)	H24C—C24—H24A	120.0
			
C6—C1—C2—C3	1.8 (3)	C13—C11—C12—C8	178.01 (12)
C20—O1—C3—C4	−2.8 (3)	C10—C11—C12—C8	0.94 (19)
C20—O1—C3—C2	177.57 (18)	C7—C8—C12—O2	0.6 (2)
C1—C2—C3—O1	178.69 (16)	C9—C8—C12—O2	178.19 (15)
C1—C2—C3—C4	−0.9 (3)	C7—C8—C12—C11	−179.34 (13)
O1—C3—C4—C5	179.97 (15)	C9—C8—C12—C11	−1.73 (19)
C2—C3—C4—C5	−0.5 (3)	C12—C11—C13—C14	179.02 (13)
C3—C4—C5—C6	1.0 (2)	C10—C11—C13—C14	−4.2 (2)
C4—C5—C6—C1	−0.2 (2)	C11—C13—C14—C15	−26.2 (2)
C4—C5—C6—C7	178.18 (14)	C11—C13—C14—C19	156.73 (15)
C2—C1—C6—C5	−1.3 (2)	C19—C14—C15—C16	−0.4 (2)
C2—C1—C6—C7	−179.72 (16)	C13—C14—C15—C16	−177.60 (12)
C5—C6—C7—C8	31.9 (2)	C14—C15—C16—C17	−0.2 (2)
C1—C6—C7—C8	−149.75 (16)	C21—O3—C17—C16	3.8 (2)
C6—C7—C8—C12	−179.27 (14)	C21—O3—C17—C18	−177.41 (14)
C6—C7—C8—C9	3.3 (2)	C15—C16—C17—O3	178.79 (13)
C10—N1—C9—C8	65.73 (14)	C15—C16—C17—C18	0.1 (2)
C22—N1—C9—C8	−171.19 (11)	O3—C17—C18—C19	−178.09 (14)
C7—C8—C9—N1	146.84 (14)	C16—C17—C18—C19	0.8 (2)
C12—C8—C9—N1	−30.56 (17)	C17—C18—C19—C14	−1.5 (3)
C9—N1—C10—C11	−66.65 (14)	C15—C14—C19—C18	1.3 (2)
C22—N1—C10—C11	170.21 (11)	C13—C14—C19—C18	178.60 (14)
N1—C10—C11—C13	−144.68 (13)	C10—N1—C22—C23	−164.76 (13)
N1—C10—C11—C12	32.13 (17)	C9—N1—C22—C23	73.26 (16)
C13—C11—C12—O2	−1.9 (2)	N1—C22—C23—C24	−122.02 (19)
C10—C11—C12—O2	−178.98 (15)		

### Hydrogen-bond geometry (Å, º)

**Table:**

*D*—H···*A*	*D*—H	H···*A*	*D*···*A*	*D*—H···*A*
C10—H10*A*···O3^i^	0.97	2.59	3.3710 (18)	138
C21—H21*C*···O2^ii^	0.96	2.54	3.466 (2)	163

Symmetry codes: (i) −*x*, −*y*+1, −*z*+1; (ii) −*x*, *y*−1/2, −*z*+1/2.
